# Identification of SNP and SSR markers in eggplant using RAD tag sequencing

**DOI:** 10.1186/1471-2164-12-304

**Published:** 2011-06-10

**Authors:** Lorenzo Barchi, Sergio Lanteri, Ezio Portis, Alberto Acquadro, Giampiero Valè, Laura Toppino, Giuseppe Leonardo Rotino

**Affiliations:** 1 DIVAPRA Plant Genetics and Breeding, University of Torino, 10095 Grugliasco, Torino, Italy; 2 CRA-ORL Research Unit for Vegetable Crops, 26836 Montanaso Lombardo, Lodi, Italy; 3 CRA-GPG Genomic Research Centre, 29017 Fiorenzuola d'Arda, Piacenza, Italy

## Abstract

**Background:**

The eggplant (*Solanum melongena *L.) genome is relatively unexplored, especially compared to those of the other major *Solanaceae *crops tomato and potato. In particular, no SNP markers are publicly available; on the other hand, over 1,000 SSR markers were developed and publicly available. We have combined the recently developed Restriction-site Associated DNA (RAD) approach with Illumina DNA sequencing for rapid and mass discovery of both SNP and SSR markers for eggplant.

**Results:**

RAD tags were generated from the genomic DNA of a pair of eggplant mapping parents, and sequenced to produce ~17.5 Mb of sequences arrangeable into ~78,000 contigs. The resulting non-redundant genomic sequence dataset consisted of ~45,000 sequences, of which ~29% were putative coding sequences and ~70% were in common between the mapping parents. The shared sequences allowed the discovery of ~10,000 SNPs and nearly 1,000 indels, equivalent to a SNP frequency of 0.8 per Kb and an indel frequency of 0.07 per Kb. Over 2,000 of the SNPs are likely to be mappable via the Illumina GoldenGate assay. A subset of 384 SNPs was used to successfully fingerprint a panel of eggplant germplasm, producing a set of informative diversity data. The RAD sequences also included nearly 2,000 putative SSRs, and primer pairs were designed to amplify 1,155 loci.

**Conclusion:**

The high throughput sequencing of the RAD tags allowed the discovery of a large number of DNA markers, which will prove useful for extending our current knowledge of the genome organization of eggplant, for assisting in marker-aided selection and for carrying out comparative genomic analyses within the *Solanaceae *family.

## Background

Eggplant (*Solanum melongena *L., 2n = 2x = 24) is a species belonging to the *Solanaceae *family. It is assumed to have been first domesticated in South and East Asia [1], and brought to Europe by Arab traders and immigrants around 600 CE [2]. In production terms, eggplant is the third most important *Solanaceae *crop species (after potato and tomato; http://faostat.fao.org), and is cultivated all over the world, but most intensively in China and India. About 2.4% of world production in 2009 is sited in Europe, with Italy being the single largest producer.

The estimated genome size of eggplant is 1.1 Gbp [3]. Knowledge of its genome organization is rather limited compared to that of either tomato or potato (http://solgenomics.net/, http://www.potatogenome.net). Genetic maps based on both inter-specific [4,5] and intra-specific [6-9] crosses have been developed. The most recent inter-specific map [5] is constituted of 347 COS and RFLP markers spanning 1,535 cM, while the most recent intra-specific maps were constructed by Barchi *et al. *[9] and Nunome *et al. *[8] and comprise 238 markers, spanning 718.7, and 236 markers, spanning 951.4 cM, respectively. Nevertheless the level of marker saturation is still low in the context of both fine mapping and genomic synteny. A small set of SSR markers was developed by Stagel *et al. *[10] from genic DNA sequence lodged in public access databases, while Nunome *et al. *[7] reported the identification of over 1,000 SSR markers from a screen of enriched gDNA and cDNA libraries. Many of these latter proved informative for intra-specific mapping and have been used to generate what is currently the best available genetic linkage map. More recently, Fukuoka *et al. *[11] have published a dataset containing a large number (*~*16,000) of transcript sequences, but these have yet to be mined for either SSR or SNP markers.

The so-called "Restriction-site Associated DNA" (RAD) method was proposed by Miller *et al. *[12] as providing a reliable means for genome complexity reduction. The concept is based on acquiring the sequence adjacent to a set of particular restriction enzyme recognition sites. The application of high throughput sequencing technology has allowed significant progress in developing a RAD genotyping platform [13]; specifically, large volumes of polymorphism data can be now generated by applying massively parallel sequencing and multiplexing with RAD tag libraries.

In this report we describe the generation of genomic RAD tags from the two parents of an F_2 _segregating population used to generate an intra-specific eggplant genetic map [9]; the RAD tags were sequenced using the Illumina platform and then annotated/categorized. These data allowed the discovery of a large number of SNP, indel and SSR markers, and some of the SNPs have been tested against a panel of eggplant accessions.

## Results and Discussion

### Sequencing and contig assembly

**Figure 1:**
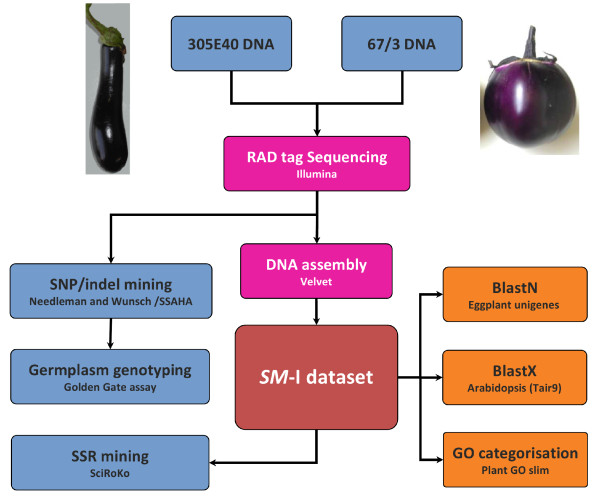
**Sequencing and gene annotation pipeline for eggplant RAD tags**.

The sequencing procedure (Figure 1) generated 10.90 million reads for '305E40' and 12.12 million for '67/3', parents of an F_2 _intra-specific mapping population (see methods section), equivalent to ~13.3 Mb of sequence for '305E40' and 13.8 Mb for '67/3'. After editing/trimming, *~*17.5 Mb high quality sequence was available. Raw data have been made available through the Sequence Read Archive (SRA) repository at NCBI (SRA035360.1). The reads were assembled into 77,876 contigs (38,935 from '305E40', 38,941 from '67/3'); the '305E40' assemblies were of mean length 351 bp (range: 218-585 bp; N50: 362 bp), and those from '67/3' of mean length 368 bp (range: 218-579 bp; N50: 382 bp). The *SM-I *(*Solanum melongena*-Illumina) dataset finally comprised 45,390 sequences, including 31,635 sequences (12.5 Mbp) in common between the two mapping parents (Table 1), and formed the basis of the subsequent annotation and functional categorization (Additional file 1). The *SM-I *dataset was also screened for the occurrence of repetitive elements. About 6.7% (1.1 Mbp) of the sequence database showed some similarity with known plant mobile elements and was thus filtered out for SNP mining procedures.

**Table 1:** Summary statistics of the RAD tags sequencing via Illumina (San Diego, CA)

Feature	305E40	67/3
Illumina reads (million)	10.90	12.12
Mb of sequences	13.30	13.82
Total Mb after sequence editing	17.50	
Contigs	38,935	38,941
Average contig length (bp)	351	368
N50^1 ^(bp)	362	382
Contig length range (bp, min-max)	218-585	218-579
Singlets	6,912	6,843
Common Contigs between parents	31,635	
Number of sequences with SNPs	5,174	
Total SNPs (frequency)	10,089 (1/1,241 bp)	
Total InDels (frequency)	874 (1/14,325 bp)	

^**1 **^N50: weighted median statistic such that 50% of the entire assembly is contained in the number of contigs equal to or greater than this value

### Sequence annotation

In all, 6,411 sequences (14.1%) of the *SM-I *dataset matched 4,761 entries in the Fukuoka 16 K eggplant annotated unigene dataset (later referred as 16 K) [11]. A BlastN search of the SGN Cornell unigene database (http://solgenomics.net/) produced significant hits from 9,476 (20.9%) of the *SM-I *sequences matching 8,244 SGN unigenes, of which ~47% originated from tomato, ~38% from potato, and ~11% from tobacco. Combining the 16 K and SGN hits produced 12,315 unique sequences; a total of 9,976 sequences were properly annotated, of which 2,123 were annotated in both the SGN and 16 K databases, 6,440 only in SGN, and 1,413 only in 16 K. Some 35,414 *SM-I *sequences were unrepresented in either of these two databases, and these were used as a batch BlastX query against the TAIR9 *Arabidopsis thaliana *protein database to allow a putative assignment of function. In all, 2,798 sequences (7.9%) produced a hit with an E value of < e^-15^, corresponding to 1,853 *A. thaliana *genes. This rather small number of hits presumably reflects sequence divergence between eggplant and *A. thaliana *orthologs, although it has been recognized that the BLAST algorithm can be rather inefficient in identifying homologous sequences when short reads are involved [14]. Globally, therefore, the *SM-I *dataset consists of some 12,774 annotated sequences which match 7,191 *A. thaliana *loci (Additional file 2).

### GO categorization

**Figure 2:**
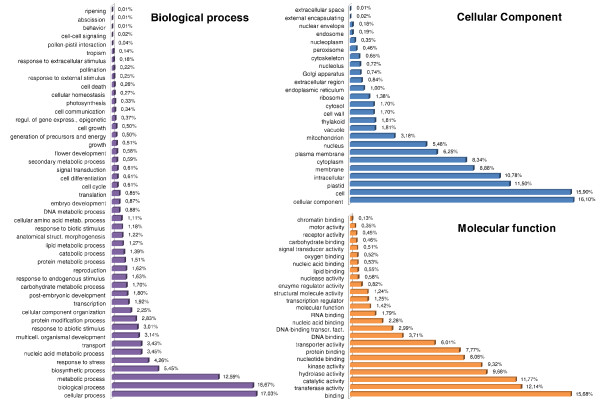
**GO term representation (%) of eggplant genomic sequences according to the three high level GO categories**.

The annotated *SM-I *sequences were functionally assigned using their *A. thaliana *orthologs as input (AGI codes) (Additional file 2), these functions were then arranged into GO slim categories (Figure 2) [15]. Since a given gene product can be associated with more than one GO term, the total number of GO terms exceeded that of the unigenes [14,16]. The eggplant *SM-I *sequences resolved into 24,522 GO terms associated with "biological process", 15,137 with "cellular component" and 12,144 with "molecular function". The "response to biotic stimulus" category applied to 492 sequences (290 GO terms), among which the majority was related to the defense response against bacterial (22.1%), nematode (10.3%) and fungal (9.3%) infection. These sequences, especially the fungal response ones, are of particular interest, as '305E40' carries a major gene conferring resistance to *Fusarium oxysporum *f. sp. *melongenae *[9,17]. Among the "response to abiotic stimulus" sequences (937 sequences, 737 GO terms), 19.5% were associated with the response to salinity stress, 12.3% to low temperature and 7.7% to high temperature. Globally, the sequences were assigned to a wide range of gene ontology categories, indicating that a wide representation of transcripts was originally present in the RAD tags. Since about 12,000 *SM-I *eggplant sequences were annotated, it seems plausible to assume that we were able to capture a consistent fraction of the eggplant gene space.

### SNP identification

**Figure 3:**
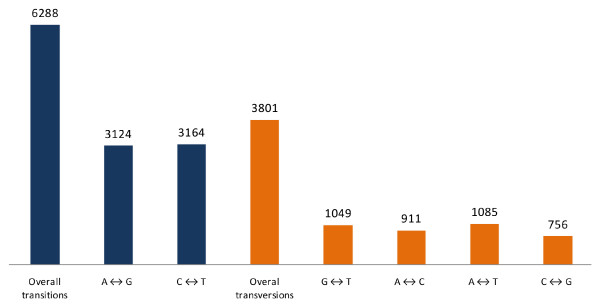
**Transitions and transversions occurring within a set of > 10,000 eggplant SNPs**.

Just over 10,000 SNPs were identified between the mapping parents, involving 5,179 of the 31,635 shared sequences (later referred as 10 K, Additional file 3), as well as 874 indels (Table 1). To minimize false positives with respect to the SNPs, paired-end reads and SNP calling based on deep multiple alignment (minimum 6x coverage) were applied. The global inter-samples SNP frequency was 0.8 per Kb, and the indel frequency 0.07 per Kb. We report the current SNP frequency as mostly belonging to the un-transcribed portion of the eggplant genome since we adopted two endonucleases recognizing GC-rich sites, being one methylation sensitive (*Sgr*AI). This SNP frequency is lower than has been detected in potato (11.5 per Kb; [18]), grapevine (2.5 per Kb in coding and 5.5 in non-coding sequence; [19]), barley (6.3 per Kb in coding sequence; [20]), maize (8.9 per Kb in coding sequence; [20]) and *Citrus *spp. (6.1 per Kb; [21]), but is similar to that found in tomato (0.6 per Kb; [22]), sweet pepper (1.0 per Kb; [23]), rice (1.7 per Kb; [24]) and confirmed the low level of intra-specific genetic polymorphism previously observed in eggplant [9]. As pointed out by Schneider *et al. *[25], however, inter-specific comparisons of SNP frequency are problematic, given that polymorphism is germplasm-, genomic context- and mating system-dependent. About two thirds of the SNPs proved to be transitions (Figure 3), which have generally been found to be the predominant type [23,25-27]. The transition/transversion ratio has been suggested to be high in a situation where a low level of genetic divergence applies, decreasing as the genetic distance between the comparator genomes rises [28,29]. The relatively high ratio of 1.65 probably therefore reflects the overall low level of polymorphism between the two mapping parents, as is generally the case within the cultivated gene pool of eggplant [10]. A rather high frequency of C/T alleles was observed, as also noted for bean [30], maize [27] and *Citrus *spp. [21,31]. In about 25% of the SNP loci, there was no additional sequence variation in either the upstream or the downstream 60 bp and almost all of them (2,354 out of 2,435) were associated with a quality score > 0.4 (the minimum threshold for the GoldenGate assay) and 2,201 produced a score of > 0.6. The identification of > 10,000 potential SNPs is clearly a major advance for eggplant genotyping; incorporation of a sample of the 2,354 high quality SNPs into a GoldenGate assay would certainly saturate the '305E40' × '67/3' linkage map, while many of the remaining ~8,000 SNPs could be assayed by other technologies, such as the Affymetrix SNP chip or the High Resolution Melting technique [32].

The successful identification of a large number of SNP (and indel and SSR) markers highlights the utility of the RAD approach for uncovering genome-wide polymorphisms, especially in materials with low polymorphism [33]. The versatility of the method lies in the ease with which different samples of the genome can be accessed merely by changing the identity of the restriction enzyme(s) used to cleave the genomic DNA; its particular advantage in the context of SNP discovery lies in the ease of aligning short DNA fragments between contrasting templates. Note also that the application of Illumina sequencing allowed for the identification of polymorphic sites outside of the restriction enzyme recognition site [13].

### Genetic diversity revealed by SNP markers

**Figure 4:**
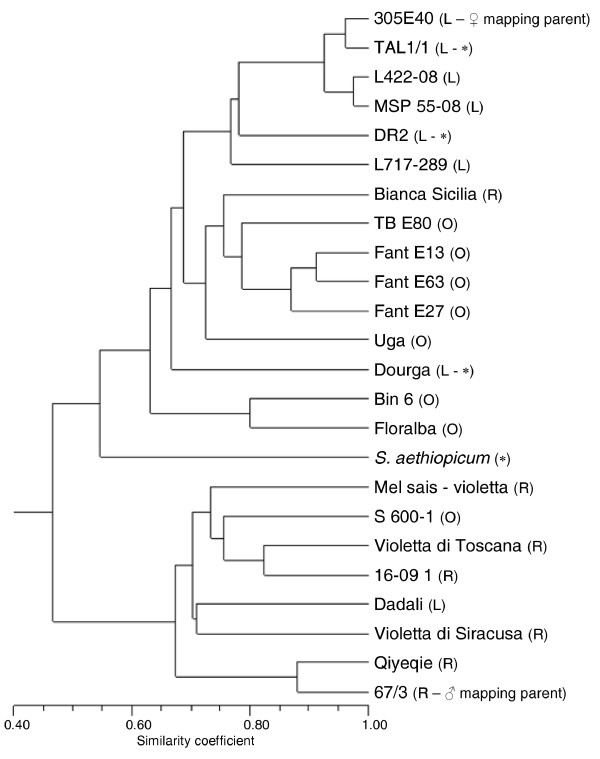
**Phylogeny of 23 *Solanum melongena *genotypes along with one accession of *S. aethiopicum***. Genotyping was based on allele calls at 343 SNP loci. In brackets: fruits shapes (L: long; O: oval; R: round) of eggplant genotypes and indication of the parental lines of the F_2 _mapping population [9]. Asterisks indicate the progenitors of the '305E40' parental line.

A sample of 384 of the 2,201 highest quality SNPs (score > 0.6) was assembled into a GoldenGate assay, which was then applied to genotype 23 *S. melongena *templates (Table 2), a representative panel of eggplant germplasm which captured a large part of variation with respect to fruit shape and colour (including '305E40' and '67/3'), together with one accession of *S. aethiopicum*. Of these, 343 produced non- ambiguous data, a percentage in agreement with that previously reported in maize [34] and soybean [35]. The two duplicated genotypes included as internal controls gave consistent calls, indicating that the assay was highly robust. The frequency of missed calls was ~ 0.6% among the eggplant templates, but was 16.0% for the *S. aethiopicum *template. PIC values ranged from 0.29 to 0.5 (mean 0.43), with 240 of the markers producing a PIC value > 0.4, a level which is suitable for genetic diversity analyses. The phylogeny of the germplasm accessions based on these SNPs suggested the presence of two major clades (Figure 4); one included '305E40' together with its progenitors 'Dourga', 'Tal1/1', 'DR2' and *S. aethiopicum*, while the second included '67/3'. Within each of these major clades, a number of sub-clades correlated with fruit shape could be recognized. Thus, the phenotypic divergence between the pair of mapping parents appears to be representative of the genetic variation present within the cultivated gene pool.

**Table 2:** *Solanum melongena *lines genotyped with SNP markers (shape and skin colour are indicated)

Genotype	Fruit type
305E40	Long dark purple
DR2	Long dark purple
TAL1/1	Long dark purple
MSP 55-08	Long dark purple
L422-08	Long dark purple
L717-289	Long dark purple
Dadali	Long light purple
Dourga	Long white
TB E80	Oval dark purple
Fant E13	Oval dark purple
Fant E27	Oval dark purple
Fant E63	Oval dark purple
Uga	Oval dark purple
Bin 6	Oval green
S 600-1	Oval purple
Floralba	Oval white
16-09 1	Round dark purple
67/3	Round violet
Qiyeqie	Round violet
Mel sais (violetta)	Round violet
Violetta di Siracusa	Round violet
Violetta di Toscana	Round violet
Bianca Sicilia	Round white

### Identification of SSRs

A screen of the *SM-I *dataset resulted in the identification of 1,797 sequences containing 1,877 putative SSRs. A small number of these SSRs (22) were discarded as they had already been previously identified [7,10,11]. The SSR was present in both mapping parents for 1,145 sequences, in '305E40' alone for 381 sequences, and in '67/3' alone for 329 sequences. At least 1,119 sequences permitted the design of PCR primers, leading to the generation of 1,155 putative markers (Additional file 4). About 4.1% of the *SM-I *sequences contained an SSR (equivalent to a density of one SSR per 9.0 Kb), which is comparable to the success rate recorded from ESTs of eggplant [7,10] and tomato [36], somewhat higher than in potato [36] but lower than in either coffee [36] or sweet pepper [36,37]. Thus the RAD technique appears to offer an effective means of discovering SSRs, especially given the understanding that SSRs are more common in transcribed rather than in genomic sequences [38].

The most abundant repeat motif among the RAD SSRs were trinucleotides (34.6%), followed by dinucleotides (18.6%) and pentanucleotides (16.6%) (Table 3), consistent with the observations of Stagel *et al. *[10]. The most common di- and tri-nucleotide motifs were AT (9.6%) and AAC (19.0%) (Table 4), in contrast to the observation in a previous study, where AG and AAG were the predominant motifs [10,14,36-39]. On the other hand Shirasawa *et al. *[40] showed that among tomato genomic SSRs, AAT is the most abundant trinucleotide and AT the most abundant dinucleotide motif, while in wheat, AAC is the predominant trinucleotide SSR motif [41]. SSRs composed of either AGG and CCG repeats were rather rare, as reported by Stagel *et al. *[10] in eggplant but also in other recent studies on *Epimidium sagittatum *[14] and *Vigna radiata *[42]. These particular motifs are relatively uncommon in dicotyledonous plant genomes [14,36], although they do feature in monocotyledonous ones [38,43]. Among the 160 mononucleotide SSRs detected, 151 were A/T; these loci have been suggested as providing a means of filling gaps in linkage maps constructed with higher order SSRs [36].

**Table 3:** The 1,855 SSR motifs identified in 1,777 sequences

Motif	Counts	%	Average motif length	Number of repeats								
				
				3	4	5	6	7	8	9	10	> 10
Mononucleotide	160	8.6	17.73	-	-	-	-	-	-	-	-	160
Dinucleotide	345	18.6	19.46	-	-	-	-	-	107	67	47	124
Trinucleotide	641	34.6	19.11	-	-	250	177	109	51	18	16	20
Tetranucleotide	216	11.6	18.44	-	125	52	21	14	2	1	-	1
Pentanucleotide	308	16.6	17.4	211	75	15	5	2	-	-	-	-
Hexanucleotide	185	10.0	23.06	85	57	29	11	2	1	-	-	-

**Table 4:** Frequencies and repeat numbers for the 20 most present SSR motifs

SSR motif	Counts	Average length	Counts/Mbp	% of the Total
AAC	352	18.21	20.77	19.0
AT	179	19.09	10.53	9.6
A	151	17.66	8.88	8.1
AG	98	20.36	5.77	5.3
AAG	95	19.55	5.59	5.1
AAAT	90	18.53	5.29	4.8
AAT	87	21.78	5.12	4.7
AAAAT	77	17.83	4.53	4.1
AC	68	19.15	4.0	3.7
AAAAG	41	18.34	2.41	2.2
ATC	37	20.08	2.18	2.0
AAAG	36	18.56	2.12	1.9
ACC	26	18.85	1.53	1.4
AAAAAT	22	22.91	1.29	1.2
AAATT	21	17.62	1.24	1.1
AATAT	20	16.75	1.18	1.1
AGG	18	20.67	1.06	1.0
AAAAC	18	17.0	1.06	1.0
ATAC	17	20.71	1.0	0.9

## Conclusions

The RAD method was highly successful for the rapid and large-scale discovery of DNA markers, even in a species recognized to be low polymorphic. Applied to a pair of eggplant mapping parents, the approach was able to define over 10,000 SNPs, 1,600 indels and 1,800 putative SSRs. The current eggplant genetic maps are far from saturated, and as such have had little impact on breeding. The early maps were based on a wide cross, as this was considered necessary to achieve a sufficient level of polymorphism for the markers then available. With the rapid advances being made in sequencing technology, it is now possible to work with intra-specific crosses which are more relevant to the breeder. The present study has generated a large number of SNP, indel and SSR assays, which should permit the rapid saturation of the best available intra-specific genetic map [9].

Our primary goal was the identification of SNP markers, however data from RAD tags sequencing made it also possible the identification of SSR motifs and respective primers pairs for their amplification. The multi-allelic SSR markers are currently widely applied for both genetic mapping and diversity analyses, despite their cost for development and their limited throughput capabilities [44]. During the last few years the exploitation of publicly available EST sequences leaded to the identification of several thousands of new SSRs markers in a wide range of vegetables species like tomato, pepper, globe artichoke, Brassica, as well as eggplant [10,36,37,39,44,45]

The GoldenGate SNP array was highly robust for *S. melongena *germplasm, but also has potential for a wide-cross population as 84% of the loci were scorable in a contrast between cultivated eggplant and its relative *S. aethiopicum*. Since these DNA markers define a specific position in the eggplant genome, they should be useful for merging the various genetic linkage maps currently available, some of which include loci related to important agronomic traits. Finally, the markers are very informative for the analysis of genetic diversity, as well as for comparative studies across species within the *Solanaceae *family.

## Methods

### Plant materials and DNA isolation

DNA was extracted from the two eggplant lines '305E40' and '67/3', which are the parents of an F_2 _intra-specific mapping population [9]. The female parent, double-haploid line '305E40', produces long, highly pigmented dark purple fruit. The parent '305E40' is an introgression line derived from the somatic hybrid *S. melongena *cv. 'Dourga'(+)*S. aethiopicum *[46] which was backcrossed with a tetraploid plant of the eggplant line 'DR2' and then subjected to anther culture; an anther-derived dihaploid plant was backcrossed 4 times with the line 'Tal1/1', then selfed two times and, finally, made completely homozygous through anther culture [17,44]. The male parent, line '67/3', was an F_8 _selection from the intra-specific cross cvs. 'Purpura' × 'CIN2'. Its fruit is round and violet coloured. The DNAs extracted from a set of 23 accessions (including the two mapping parents) representative of the *S. melongena *gene pool (Table 2), together with an accession of *S. aethiopicum *(a progenitor of '305E40') were tested with a subset of the newly developed SNP assays. All DNA samples were extracted from young leaves, using the GenElute™ Plant Genomic DNA Miniprep kit (Sigma, St. Louis, MO), following the manufacturer's protocol.

### RAD library preparation, sequencing, assembly

The RAD library was constructed at Floragenex Inc. (USA), according to the protocol described by Baird *et al. *[13], as follows. Genomic DNA (300 ng) was digested for 60 min at 37° C in a 50 μL reaction containing 20 U each of *SgrA*I and *Pst*I (New England Biolabs, Beverly MA, USA). The reactions were stopped by holding at 65° C for 20 min. The P1 adapter (a modified Illumina adapter, see Baird *et al. *[13] was ligated to the products of the restriction reaction, and the "barcoding" of the various samples was achieved with a set of index nucleotides in the P1 adapter sequence. A 2.5 μL aliquot of 100 nM P1 adapter was added to each sample, along with 1 μL 10 mM ATP (Promega), 1 μL 10 × NEB Buffer4, 1 μL (equivalent to 1,000 U) T4 DNA ligase (Enzymatics, Inc) and 5 μL water, and the reaction was incubated at room temperature for 20 min, and then heat-inactivated (20 min at 65° C). The reactions were then pooled and the products randomly sheared to a mean size of 500 bp using a Bioruptor (Diagenode). The material was electrophoresed through a 1.5% agarose gel, and the DNA in the range 300-800 bp isolated using a MinElute Gel Extraction Kit (Qiagen). The dsDNA ends were treated with end blunting enzymes (Enzymatics, Inc) to remove overhangs, and the samples purified by passing through a MinElute column (Qiagen). 3'-adenine overhangs were then added by the addition of 15 U Klenow exo- (Enzymatics), followed by an incubation at 37° C for 10 min. Following re-purification, 1 μL 10 μM P2 adapter (a modified Illumina adapter, see Baird *et al. *[13]) was ligated, as described above for P1. The samples were then purified as above, and eluted in a volume of 50 μL. Following quantification (Qubit fluorimeter), 20 ng were taken as the template for a 100 μL PCR containing 20 μL Phusion Master Mix (NEB), 5 μL 10 μM P1 adapter primer (Illumina), 5 μL 10 μM P2 adapter primer (Illumina) and water. The Phusion PCR settings followed product guidelines (NEB) over 18 cycles. The amplicons were gel purified, the size range 300-700 bp was excised from the gel, its DNA content adjusted to 3 ng/μL. RADs from each parent were sequenced on a Genome Analyzer II (Illumina, San Diego, CA) using paired end 54 bp sequence reads. The paired end sequences from each parent were pooled and segregated by single read RAD sequences. Velvet [47] was used to assemble consensus LongRead contigs from the paired end data. Repetitive element occurrence was searched via CENSOR, a software tool which screens query sequences against a reference collection of repeats (http://www.girinst.org/censor; [48]), adopting default parameters and considering Viridiplantae as target database.

### Sequence annotation

CAP3 [49] algorithm was used to identify sequences in common between the mapping parents using default parameters with some modifications (overlap length cut-off = 80 and overlap percent identity cut-off = 95). The resulting dataset (*SM-I; Solanum melongena*-Illumina) included singlets from '67/3' and '305E40' as well as contigs deriving from both RAD rounds. A stand-alone BLAST tool was used to provide the optimal annotation for each dataset.

A BlastN search was performed against the SGN Cornell unigene database (http://solgenomics.net/), using as cut-off parameters 90% identity and a minimum alignment of 100 bp. A second BlastN search was made against the 16 K Fukuoka eggplant unigene dataset (in the article referred as 16 K, http://vegmarks.nivot.affrc.go.jp[11]), using as cut-off parameters 95% identity and a minimum alignment of 100 bp. A BlastX search was carried out against the TAIR9 dataset (http://www.arabidopsis.org), adopting a threshold E-value of e^-15^. The annotated sequences were assigned a function based on the Gene Ontology tool available at TAIR (http://www.arabidopsis.org/tools/bulk/go/), using *A. thaliana *orthologs as input (AGI codes), and mapped to higher level categories (plant GO Slim) using GOSlimViewer [50] according to the three principal GO categories "molecular function", "biological process" and "cellular localization" [15].

### SNP discovery

SNPs were called using a short read alignment algorithm [51] which aligned non-assembled 50 bp Illumina reads from '67/3' against the '305E40' assembly, by analogy with the MAQ style sequence pileup [52] at a minimum coverage of 6x; to call indels, an SSAHA-based alignment strategy [53] was applied. Both SNPs and indels were regarded as true polymorphisms, when each allele was observed at least three times.

Each SNP was assigned a designability score via a dedicated "assay design tool" (http://www.illumina.com), which identified SNP loci free of other polymorphisms 60 bp either upstream or downstream. A quality score, based on the probability of good performance using the Illumina Golden Gate assay, was assigned to each SNP, where a score > 0.6 indicated a high probability of success.

### Genetic diversity assessment based on the GoldenGate assay

The GoldenGate assay (Illumina, San Diego, CA) was used for SNP genotyping at the UC Davis Genome Center. Automatic allele calling for each locus was obtained by GenCall software (Illumina). As an internal control, two duplicate templates were included in each run. An estimate of PIC (Polymorphism Information Content) was made following the suggestion of Anderson *et al. *[54]. Each SNP locus was scored in binary fashion. A co-phenetic distance matrix based on co-dominant markers was generated, as described by Smouse *et al. *[55] and used to construct a UPGMA-based dendrogram as implemented within NTSYS software package v2.10 [56].

### SSR identification

SSR motifs were identified by SciRoKo software [57]. Both perfect and imperfect mono, di-, tri-, tetra-, penta- and hexanucleotide motifs were targeted. Primer pairs were designed from the flanking sequences using PRIMER3 software [58] in batch mode, as implemented in the SciRoKo package. The target amplicon size range was set as 125-250 bp, the optimal annealing temperature 60° C, and the optimal primer length 20 bp.

## Authors' contributions

SL and GLR planned and supervised the work. LB carried out BLAST analyses, SSR primer design and sequence annotation; EP carried out the diversity analysis, AA supervised the BLAST analyses, LT and GLR provided plant materials; GV contributed to SNP identification. All the authors read and approved the final version of the manuscript.

## Supplementary Material

Additional file 1 ***SM-I *dataset**. The zip file contains 5 files (1a-1e): 1a: "add_file_1a_*SM-I*.fas" (file format: .txt - fasta file); 1b: "add_file_1b_contigs_parents_*SM-I*.txt" (file format: .txt - fasta file); 1c: "add_file_1c_singlets parent 67_3.txt" (file format: .txt - fasta file); 1d: "add_file_1d_singlets parent 305E40.txt" (file format: .txt - fasta file); 1e: "add_file_1e_contigs_parents_*SM-I*.ace" (file format: .ace; the ACE viewer "Tablet" is available at http://bioinf.scri.ac.uk/tablet). Click here for file

Additional file 2 ***SM-I *dataset annotation**. Click here for file

Additional file 3 **SNP dataset information**. Click here for file

Additional file 4 **SSR statistics and primer pairs designed**. Click here for file
